# *IL1A* polymorphisms is a risk factor for colorectal cancer in Chinese Han population: a case control study

**DOI:** 10.1186/s12885-019-5395-9

**Published:** 2019-02-28

**Authors:** Hong Ji, Le Lu, Jingjing Huang, Yang Liu, Binchao Zhang, Hui Tang, Dangze Sun, Yafei Zhang, Hao Shang, Yiming Li, Hongwei Lu

**Affiliations:** 1 Department of General Surgery, The Second Affiliated Hospital, Xi’an Jiaotong University School of Medicine, #157, West fifth Road, Xi’an, 710004 Shaanxi People’s Republic of China; 2 Department of Surgery, The People’s Hospital of Zhenba Country, Hanzhong, 723600 Shaanxi People’s Republic of China; 3 Department of General Surgery, The People’s Hospital of Shangzhou District, Shangluo, 726000 Shaanxi People’s Republic of China

**Keywords:** Single-nucleotide polymorphisms, *IL1A*, Stratification analysis, Colorectal cancer, Chinese Han population

## Abstract

**Background:**

Colorectal cancer (CRC) is one of the most common cancers worldwide, and genetic variations exert distinct roles in its pathogenesis. Single nucleotide polymorphisms (SNPs) in *interleukin 1 alpha* (*IL1A*) were reported to be correlated to the susceptibility of diverse cancers. The aim of this study was to assess the association of *IL1A* SNPs with the risk of colorectal cancer in a Chinese Han population.

**Methods:**

To evaluate the correlation between *IL1A* polymorphisms and CRC risk, Agena MassARRAY platform was used for genotype determination among 248 CRC patients and 463 controls. The relationships between *IL1A* variants and CRC susceptibility were examined by logistic regression analysis. Stratified analysis was conducted for the association detection in males and females. Haplotype construction and analysis were applied to evaluate the potential relationship between the genetic block and the risk of CRC. SNP functional exploration was performed with available bioinformatics datasets.

**Results:**

After adjusting for age and gender, the “AA” genotype of rs2856838 exhibited a risk association with colorectal cancer in the recessive model (adjusted OR = 1.98, 95% CI: 1.05–3.72, *p* = 0.036). With stratified analysis, the recessive models of rs3783550 (OR = 2.17, 95% CI: 1.03–4.60, *p* = 0.043), rs2856838 (OR = 2.58, 95% CI: 1.13–5.87, *p* = 0.024), rs1609682 (OR = 2.20, 95% CI: 1.04–4.65, *p* = 0.040), and rs3783521 (OR = 2.13, 95% CI: 1.01–4.49, *p* = 0.048) revealed significant relationships between these variants and an increased CRC risk only in females. Bioinformatics analysis also revealed the putative functions of the selected SNPs.

**Conclusions:**

This study demonstrated that rs2856838 could influence the susceptibility to CRC in Chinese Han population from northwest China. *IL1A* variants rs3783550, rs2856838, rs1609682, and rs3783521 were associated with CRC risk only in females.

**Electronic supplementary material:**

The online version of this article (10.1186/s12885-019-5395-9) contains supplementary material, which is available to authorized users.

## Background

Colorectal cancer (CRC) is one of the most common cancers worldwide, and it is the third leading cause of cancer-related deaths in both genders. In the past two decades, an increasing incidence of CRC was found in China as lifestyles and eating habits have become progressively changed [16, 33]. The etiology of CRC are multifactorial, and epidemiological evidence suggests that smoking cigarettes, unhealthy eating habits and lack of exercises are three well-known causal factors for CRC development [5, 25]. It has been confirmed that long-term exposure to these adverse habits may eventually increase the individual CRC risk, however, genetic aberrations, such as single-nucleotide polymorphisms (SNPs), play more important roles in the pathogenesis of CRC [2, 8, 23, 27, 28].

Accumulating evidence has demonstrated that most cancers develop from long-time chronic inflammation, including CRC, which could be attributed to two common inflammatory bowel diseases, namely, Crohn’s disease and ulcerative colitis [7, 18]. Inflammatory and tumour cells could produce cytokines and chemokines that facilitate tumour promotion and progression [6]. Interleukin 1 (IL-1) is a family of pleiotropic cytokine with multiple functions in human innate inflammatory and immune responses [3]. It has also been reported that IL-1 proteins are involved in tumour angiogenesis, invasion, proliferation, and metastasis [22]. Together with IL-1β, IL-1α, encoded by *IL1A* (*interleukin 1 alpha*), is defined as an “alarm cytokine” that belongs to IL-1 cluster and plays dual roles in malignant tumour progression [22]. Membrane-associated IL-1α could elevate immunogenicity of the tumour cell and exert positive effect on anti-tumour immune surveillance and tumour regression. However, secretable form of IL-1α in the microenvironment of tumour cells has been proved to facilitate tumour invasiveness and angiogenesis [21].

Furthermore, it is widely recognized that the polymorphisms of *IL-1A* is associated with diverse diseases. Gao et al. has reported an insertion/deletion (ins/del) polymorphism (rs3783553, TTCA) in *IL1A* that may contribute to hepatocellular cancer susceptibility, and revealed the regulatory function of the variation on IL1α expression by disrupting the binding sites for miR-122 and miR-378 [12]. This functional polymorphism has also been demonstrated in gastric cancer and cervical carcinoma [13, 20, 31]. Additionally, the relationships between *IL1A* polymorphisms and the risk of papillary thyroid cancer, nasopharyngeal cancer and epithelial ovarian cancer have been reported as well [11, 29, 32].

A recent work has elucidated a decreased tumour expression of *IL1A* in colorectal adenocarcinoma, which indicated the potential role of IL1A in the etiology of CRC. However, few studies have examined the associations of *IL1A* polymorphisms with the risk of CRC. In our study, we investigated the effects of five *IL1A* variants (rs3783550, rs3783546, rs2856838, rs1609682, and rs3783521) on the susceptibility to CRC, which is supposed to provide more evidence for *IL1A* in CRC pathogenesis and contribute to early CRC risk estimation among the individuals of Chinese Han ancestry.

## Methods

### Study subjects

The current research involved a total of 248 CRC patients (143 males and 105 females) and 463 controls (265 males and 198 females) with unrelated Chinese Han ancestries. All CRC cases were diagnosed and confirmed by two independent pathological examinations. As for the eligible CRC cases selection, the individuals without other types of cancer, familial adenomatous polyposis, hereditary nonpolyposis colorectal cancer or undergone previous radiotherapy and chemotherapy were included. With regard to healthy controls, the subjects who had suffered from chronic or severe endocrine and metabolic diseases, digestive system disorders and malignant diseases were excluded from this study. The controls were polyp free individuals at recruitment. Moreover, the individuals with family colorectal disease and cancer history were excluded from control group as well.

### SNP genotyping

By reading the previous publications of *IL1A* polymorphisms, we selected SNPs which could impact cancer risk, and searched the genetic data of them in dbSNP database (https://www.ncbi.nlm.nih.gov/snp/) and 1000 Genomes database (http://www.internationalgenome.org/). Only the SNPs whose minor allele frequency (MAF) beyond 5% in Asian populations were included in this study in order to achieve adequate statistical power. Finally, five candidate variations rs3783550, rs3783546, rs2856838, rs1609682, and rs3783521 in *IL1A* gene were selected for genotyping. Five millilitres venous blood was collected from the subjects when they were recruited in this study. Genomic DNA was extracted from the blood with the Whole Blood Genomic DNA Purification Kit (GoldMag, Xi’an, China). The PCR primers used in multiplexed PCR assay were designed by Agena Bioscience Assay Design Suite V2.0 software and are showed in Additional file 1: Table S1[10]. In order to improve the PCR efficiency and ensure that the amplification primers, extension primers and extension products could be differed by their molecular weight, a tag 10-base sequence (5′-ACGTTGGATG-3′) was added to the 5′ end of each amplification primer. SNP genotyping was carried out with the usage of the Agena Nanodispenser (Agena Bioscience, San Diego, USA) and MassARRAY iPLEX platform (Agena Bioscience, San Diego, USA) [10]. The procedure for SNP genotyping was described as follows. First, a first round PCR was performed to increase and concentrate the target genomic fragments containing the polymorphisms. Second, using the products obtained from the last step as the templates, only one ‘mass-modified’ nucleotide was added to the polymorphic locus at the end of the extension primer fragment in extension reaction. Third, the analyte mixture was dropped to a SpectroCHIP Array by Agena Bioscience Nanodispenser and further processed by the matrix-assisted laser desorption/ionization—time of flight (MALDI-TOF) mass spectrometry. With this method, the analytes could be differed according to their flight time in the system and the nucleotide at the each SNP locus was identified. Finally, the genotyping results were managed and presented in the Agena Bioscience TYPER version 4.0 software [10, 24].

### Statistical analyses

SPSS 18.0 software (SPSS, Chicago, IL, USA) was employed for primary statistical analysis. The heterozygosity for the five SNPs in control cohort was assessed by the Fisher’s exact test to detect their compliance with Hardy-Weinberg equilibrium (HWE). Allele and genotype frequencies of each SNP in CRC cases and healthy controls were calculated and evaluated for the difference with Pearson’s χ^2^ test. Logistic regression analysis with adjustment for age and gender was applied to the odds ratio (OR) and 95% confidence interval (CI) assessment [4]. Adjustment process is usually implemented in the statistical analysis to eliminate the influence of confounding factors, such as age and gender. In all statistical analysis, *p* ≤ 0.05 was regarded as statistical difference. The associations of the five candidate variants with the CRC risk were examined in codominant, dominant and recessive genetic models using SNPstats online software (https://www.snpstats.net/start.htm). Additionally, we used the Haploview software package, version 4.2 (http://analysis.bio-x.cn/myAnalysis.php) for linkage disequilibrium (LD) construction and haplotype evaluation [30]. Using the genotyping results, we first constructed the LD pattern among genetic variants. The status of LD was analyzed using two parameters: r^2^ and D’. And D’ = 1 is defined as complete linkage disequilibrium. The allele combination of the variants in one LD block was considered as haplotype. We selected the haplotypes with frequency greater than 5% to evaluate whether the haplotypes were associated with CRC risk. These associations were detected using SHEsis software with available OR (95% CI) and *p* value.

### Bioinformatics prediction and expression quantitative trait loci (eQTL) analysis

HaploReg v4.1 database (https://pubs.broadinstitute.org/mammals/haploreg/haploreg.php) was applied for SNP functional annotation using bioinformatics methods. Expression quantitative trait loci (eQTL) analysis were carried out with the data available on the Genotype-Tissue Expression (GTEx) project (https://gtexportal.org/home/). The impact of each candidate SNP on *IL1A* expression was determined in several human tissues and *p*-values were provided for statistical significance assessment.

## Results

### Population characteristics

The demographic information of the enrolled CRC patients and healthy controls are listed in Additional file 1: Table S2. After applicable statistical evaluation, there was no significant difference in gender observed between patients and controls. The mean age [± standard deviation (SD)] of the patient group was 58.69 ± 12.77 years at diagnosis and of the controls was 50.65 ± 11.79 years at recruitment.

### SNPs and the risk of CRC

Basic information and allele frequencies of the *IL1A* polymorphisms are presented in Table 1. None of the five SNPs was excluded because of the deviation from HWE at the 5% *p* level in the control population. The minor allele of each SNP was assumed a risk factor compared to the wild-type allele. Results of the genetic model analysis are showed in Table 2. The frequency of the homozygous “A/A” genotype of the *IL1A* SNP rs2856838 differed significantly between patients and controls (8.9% vs. 5.2%, *p* < 0.05). An association between allele “A/A” of rs2856838 and an increased risk of CRC was observed under the recessive model (adjusted OR = 1.98, 95% CI: 1.05–3.72, *p* = 0.036) after adjustments for age and gender.

**Table 1:** Basic information and allele frequencies of the five selected SNPs

SNP_ID	Gene	Chromosome	Position	Allele	Minor allele frequency		HWE *p* value	OR (95% CI)	*p* ^a^
					Case	Control			
rs3783550	IL1A	2q13	113,532,885	T/G	0.358	0.326	0.399	1.15 (0.91–1.45)	0.222
rs3783546	IL1A	2q13	113,534,830	C/G	0.353	0.325	0.458	1.13 (0.90–1.42)	0.296
rs2856838	IL1A	2q13	113,539,972	A/G	0.256	0.247	0.319	1.04 (0.81–1.35)	0.716
rs1609682	IL1A	2q13	113,540,205	T/G	0.354	0.327	0.398	1.12 (0.90–1.42)	0.298
rs3783521	IL1A	2q13	113,543,577	G/A	0.356	0.326	0.399	1.14 (0.91–1.44)	0.252

*SNP* Single nucleotide polymorphism, *HWE* Hardy-Weinberg equilibrium, *OR* Odds ratio, 95% CI, 95% confidence interval

HWE *p*-value obtained from Fisher’s exact test (*p* > 0.05)

*p*^a^ values were calculated from Pearson’s χ^2^ test regarding to the allele distribution frequencies among CRC patients and healthy controls

*p* < 0.05 indicates SNP with statistical significance

**Table 2:** Genetic model analyses of *IL1A* SNPs and the risk of CRC

SNP_ID	Genotypes	Controls, n (%)	Patients, n (%)	Without adjustment		With adjustment	
				OR (95% CI)	*p* ^a^	OR (95% CI)	*p* ^b^
rs3783550							
	G/G	206 (44.5%)	103 (41.7%)	1.00		1.00	
Codominant	G/T	212 (45.8%)	111 (44.9%)	1.05 (0.75–1.46)	0.330	1.01 (0.72–1.44)	0.310
	T/T	45 (9.7%)	33 (13.4%)	1.47 (0.88–2.44)		1.49 (0.88–2.52)	
Dominant (ref: G/G)	G/T-T/T	257 (55.5%)	144 (58.3%)	1.12 (0.82–1.53)	0.470	1.10 (0.79–1.52)	0.580
Recessive (ref: G/G + G/T)	T/T	45 (9.7%)	33 (13.4%)	1.43 (0.89–2.31)	0.140	1.48 (0.90–2.43)	0.130
rs3783546							
	G/G	206 (44.7%)	104 (41.9%)	1.00		1.00	
Codominant	G/C	210 (45.5%)	113 (45.6%)	1.07 (0.77–1.48)	0.500	1.02 (0.73–1.45)	0.500
	C/C	45 (9.8%)	31 (12.5%)	1.36 (0.82–2.28)		1.37 (0.80–2.34)	
Dominant (ref: G/G)	G/C-C/C	255 (55.3%)	144 (58.1%)	1.12 (0.82–1.53)	0.480	1.09 (0.78–1.51)	0.620
Recessive (ref: G/G + G/C)	C/C	45 (9.8%)	31 (12.5%)	1.32 (0.81–2.15)	0.270	1.35 (0.82–2.24)	0.240
rs2856838							
	G/G	257 (55.8%)	143 (57.7%)	1.00		1.00	
Codominant	G/A	180 (39%)	83 (33.5%)	0.83 (0.60–1.15)	0.096	0.83 (0.58–1.17)	0.063
	A/A	24 (5.2%)	22 (8.9%)	1.65 (0.89–3.04)		1.84 (0.96–3.50)	
Dominant (ref:G/G)	G/A-A/A	204 (44.2%)	105 (42.3%)	0.93 (0.68–1.26)	0.620	0.94 (0.68–1.31)	0.720
Recessive (ref: G/G + G/A)	A/A	24 (5.2%)	22 (8.9%)	1.77 (0.97–3.23)	0.064	**1.98 (1.05–3.72)**	***0.036***
rs1609682							
	G/G	205 (44.4%)	105 (42.5%)	1.00		1.00	
Codominant	G/T	212 (45.9%)	109 (44.1%)	1.00 (0.72–1.40)	0.350	0.97 (0.68–1.37)	0.310
	T/T	45 (9.7%)	33 (13.4%)	1.43 (0.86–2.38)		1.45 (0.85–2.45)	
Dominant (ref: G/G)	G/T-T/T	257 (55.6%)	142 (57.5%)	1.08 (0.79–1.47)	0.630	1.05 (0.76–1.46)	0.770
Recessive (ref: G/G + G/T)	T/T	45 (9.7%)	33 (13.4%)	1.43 (0.89–2.31)	0.150	1.47 (0.89–2.42)	0.130
rs3783521							
	A/A	206 (44.5%)	103 (41.7%)	1.00		1.00	
Codominant	G/A	212 (45.8%)	112 (45.3%)	1.06 (0.76–1.47)	0.400	1.02 (0.72–1.44)	0.410
	G/G	45 (9.7%)	32 (13.0%)	1.42 (0.85–2.37)		1.42 (0.84–2.42)	
Dominant (ref: A/A)	G/A-G/G	257 (55.5%)	144 (58.3%)	1.12 (0.82–1.53)	0.470	1.09 (0.79–1.51)	0.610
Recessive (ref: A/A + G/A)	G/G	45 (9.7%)	32 (13.0%)	1.38 (0.85–2.24)	0.190	1.41 (0.86–2.33)	0.180

*SNP* Single nucleotide polymorphism, *OR* Odds ratio; 95% CI: 95% confidence interval

*p*^a^-values were calculated by logistic regression analysis with the comparison between CRC patients and healthy controls

*p*^b^-values were calculated by logistic regression analysis with adjustments for age and gender

Bold italics indicates the SNP with statistical significance (*p* < 0.05)

In accordance with the stratified analysis by gender, we found that the *IL1A* variants rs3783550, rs2856838, rs1609682 and rs3783521 did not show any significant association with CRC risk in males, whereas the statistical significance was detected in females (Table 3). The frequency of homozygous “T/T” genotype of *IL1A* SNPs rs3783550 and rs1609682 differed significantly between patients and controls in females (rs3783550: 15.2% vs. 8.1%; rs1609682: 15.4% vs. 8.1%, *p* < 0.05), respectively. Moreover, rs3783550 and rs1609682 were associated with an increased risk of CRC based on the results of the recessive model (rs3783550: OR = 2.17, 95% CI: 1.03–4.60, *p* = 0.043; rs1609682: OR = 2.20, 95% CI: 1.04–4.65, *p* = 0.040). The frequency of homozygous “A/A” genotype of rs2856838 differed significantly between patients and controls in females (13.3% vs. 6.1%, *p* < 0.05) as well. The recessive genetic model also provided the evidence for the role of rs2856838 in CRC susceptibility among Chinese Han women (OR = 2.58, 95% CI: 1.13–5.87, *p* = 0.024). Additionally, rs3783521 homozygous genotype “G/G” distributed differently between the case and control group (15.2% vs. 8.1%, *p* < 0.05). Further genetic model analysis revealed that SNP rs3783521 was linked to an increased risk of CRC in females according to the recessive model (OR = 2.13, 95% CI: 1.01–4.49, *p* = 0.048). However, no statistically significant association was detected between SNP rs3783546 and CRC risk in Chinese Han women.

**Table 3:** Association of the selected SNPs in *IL1A* with CRC risk after stratified analysis by gender

SNP_ID	Genotypes	Male		OR (95% CI)	*p* ^a^	Female		OR (95% CI)	*p* ^b^
		Controls, n(%)	Patients, n(%)			Controls, n(%)	Patients, n(%)		
rs3783550									
	G/G	113 (42.6%)	62 (43.7%)	1.00		93 (47.0%)	41 (39.0%)	1.00	
Codominant	G/T	123 (46.4%)	63 (44.4%)	0.85 (0.53–1.38)	0.800	89 (45.0%)	48 (45.7%)	1.22 (0.73–2.04)	0.096
	T/T	29 (10.9%)	17 (12.0%)	0.96 (0.47–1.98)		16 (8.1%)	16 (15.2%)	2.41 (1.09–5.34)	
Dominant (ref: G/G)	G/T-T/T	152 (57.4%)	80 (56.3%)	0.88 (0.56–1.38)	0.560	105 (53.0%)	64 (61.0%)	1.40 (0.86–2.27)	0.180
Recessive (ref: G/G + G/T)	T/T	29 (10.9%)	17 (12.0%)	1.05 (0.53–2.06)	0.900	16 (8.1%)	16 (15.2%)	***2.17 (1.03–4.60)***	***0.043***
rs3783546									
	G/G	113 (42.8%)	62 (43.4%)	1.00		93 (47.2%)	42 (40.0%)	1.00	
Codominant	G/C	122 (46.2%)	65 (45.5%)	0.87 (0.54–1.40)	0.850	88 (44.7%)	48 (45.7%)	1.21 (0.72–2.01)	0.170
	C/C	29 (11.0%)	16 (11.2%)	0.92 (0.44–1.90)		16 (8.1%)	15 (14.3%)	2.18 (0.97–4.85)	
Dominant (ref: G/G)	G/C-C/C	151 (57.2%)	81 (56.6%)	0.88 (0.56–1.38)	0.580	104 (52.8%)	63 (60.0%)	1.35 (0.83–2.20)	0.220
Recessive (ref: G/G + G/C)	C/C	29 (11.0%)	16 (11.2%)	0.98 (0.49–1.95)	0.960	16 (8.1%)	15 (14.3%)	1.98 (0.93–4.21)	0.080
rs2856838									
	G/G	142 (54.0%)	88 (61.5%)	1.00		115 (58.1%)	55 (52.4%)	1.00	
Codominant	G/A	109 (41.4%)	47 (32.9%)	0.68 (0.42–1.10)	0.260	71 (35.9%)	36 (34.3%)	1.06 (0.63–1.77)	0.078
	A/A	12 (4.6%)	8 (5.6%)	1.07 (0.39–2.94)		12 (6.1%)	14 (13.3%)	2.63 (1.13–6.15)	
Dominant (ref: G/G)	G/A-A/A	121 (46.0%)	55 (38.5%)	0.72 (0.46–1.14)	0.160	83 (41.9%)	50 (47.6%)	1.27 (0.79–2.06)	0.330
Recessive (ref: G/G + G/A)	A/A	12 (4.6%)	8 (5.6%)	1.24 (0.46–3.36)	0.670	12 (6.1%)	14 (13.3%)	***2.58 (1.13–5.87)***	***0.024***
rs1609682									
	G/G	113 (42.6%)	64 (44.8%)	1.00		92 (46.7%)	41 (39.4%)	1.00	
Codominant	G/T	123 (46.4%)	62 (43.4%)	0.81 (0.50–1.30)	0.670	89 (45.2%)	47 (45.2%)	1.18 (0.70–1.98)	0.100
	T/T	29 (10.9%)	17 (11.9%)	0.93 (0.45–1.90)		16 (8.1%)	16 (15.4%)	2.39 (1.08–5.31)	
Dominant (ref: G/G)	G/T-T/T	152 (57.4%)	79 (55.2%)	0.83 (0.53–1.31)	0.420	105 (53.3%)	63 (60.6%)	1.36 (0.83–2.21)	0.220
Recessive (ref: G/G + G/T)	T/T	29 (10.9%)	17 (11.9%)	1.03 (0.53–2.04)	0.920	16 (8.1%)	16 (15.4%)	***2.20 (1.04–4.65)***	***0.040***
rs3783521									
	A/A	113 (42.6%)	62 (43.7%)	1.00		93 (47.0%)	41 (39.0%)	1.00	
Codominant	G/A	123 (46.4%)	64 (45.1%)	0.86 (0.53–1.38)	0.820	89 (45.0%)	48 (45.7%)	1.22 (0.73–2.04)	0.110
	G/G	29 (10.9%)	16 (11.3%)	0.92 (0.44–1.90)		16 (8.1%)	16 (15.2%)	2.36 (1.07–5.22)	
Dominant (ref: A/A)	G/A-G/G	152 (57.4%)	80 (56.3%)	0.87 (0.55–1.37)	0.540	105 (53.0%)	64 (61.0%)	1.39 (0.86–2.26)	0.180
Recessive (ref: A/A + G/A)	G/G	29 (10.9%)	16 (11.3%)	0.99 (0.50–1.97)	0.980	16 (8.1%)	16 (15.2%)	***2.13 (1.01–4.49)***	***0.048***

*SNP* Single nucleotide polymorphism, *OR* Odds ratio; 95% CI: 95% confidence interval

*p*^a^-values were calculated by logistic regression analysis with adjustment for age in males

*p*^b^-values were calculated by logistic regression analysis with adjustment for age in females

Bold italics indicates the SNP with statistical significance (*p* < 0.05)

### CRC and haplotypes on chromosome 2q13

**Fig. 1:**
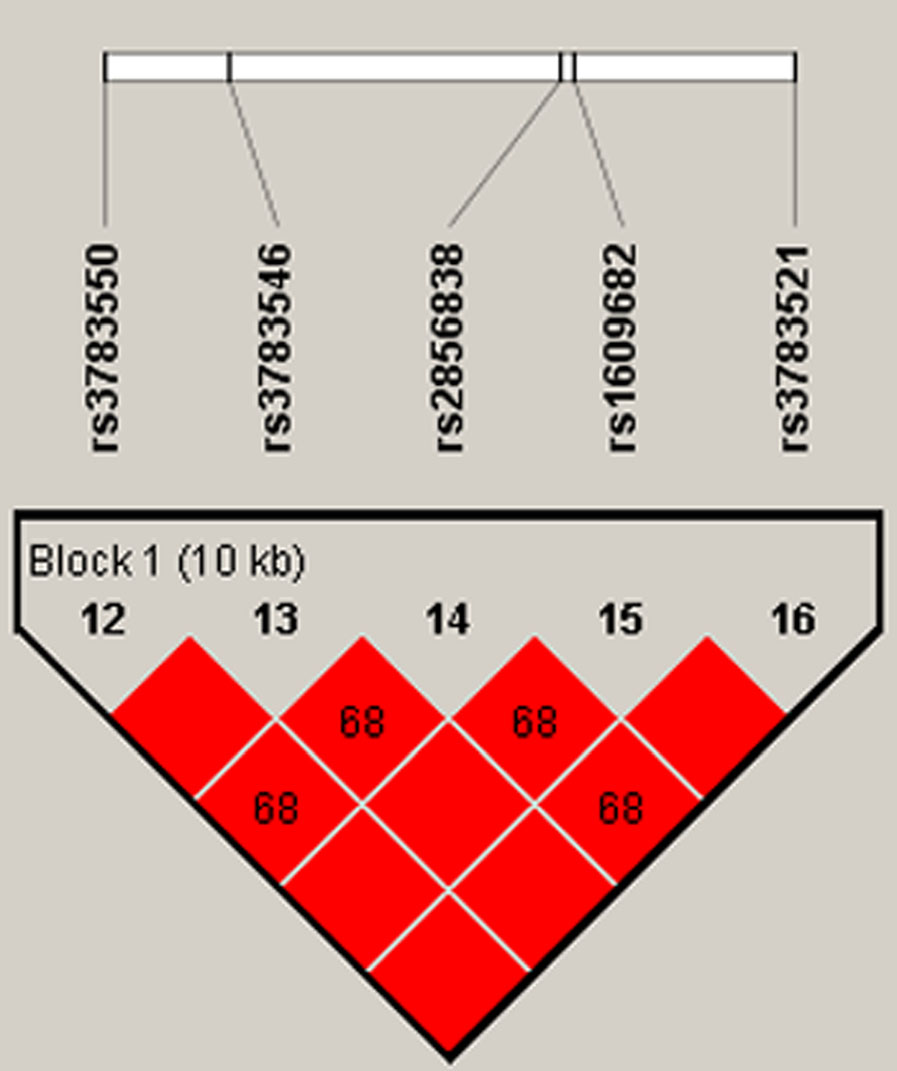
Haplotype block map formed by the SNPs in *IL1A* gene. Haplotype block are formed by rs3783550, rs3783546, rs2856838, rs1609682 and rs3783521 at 10 kb totally

Finally, five *IL1A* polymorphisms (rs3783550, rs3783546, rs2856838, rs1609682, rs3783521) were mapped to a 10-kb LD block and displayed three haplotypes with frequencies of more than 5% in our subjects (Table 4). In Fig. 1, the red squares of the *IL1A* LD block represented significant linkage between the five SNPs. The LD degree is displayed by standard color schemes with bright red for very strong LD (D’ = 1). According to the haplotype analysis, there were no statistically significant differences appearing between patients and controls among any of the *IL1A* haplotype frequencies.

**Table 4:** *IL1A* haplotype frequencies and the association with CRC risk

Block	Haplotype	Freq (case)	Freq (control)	OR (95% CI)	*p* ^a^
rs3783550/rs3783546/rs2856838 /rs1609682/rs3783521	GGGGA	0.640	0.674	1.00	
	TCATG	0.250	0.249	1.07 (0.83–1.39)	0.600
	TCGTG	0.100	0.077	1.33 (0.90–1.97)	0.150

*OR* Odds ratio; 95% CI: 95% confidence interval

*p*^a^-values were obtained after adjustments for gender and age with the comparison between CRC patients and healthy controls

### SNP functional evaluation

In order to evaluate the possible function harbored by the five selected variants, we performed a bioinformatics analysis using HaploReg v4.1 database. All the variations were successfully predicted as regulatory SNPs with different biological functions (Additional file 1: Table S3). Furthermore, eQTL analysis uncovered remarkable relationships between these SNPs and *IL1A* expression in skin, testis or pituitary, which indicated the effects of different genotypes on gene expression (Additional file 1: Table S4).

## Discussion

In this study, we genotyped five polymorphisms of *IL1A* and evaluated their correlations with the risk of CRC in a Chinese Han population. Our results first showed that four SNPs (rs3783546, rs2856838, rs1609682, rs3783521) were associated with CRC susceptibility in Chinese Han females.

Interleukin-1 alpha (IL-1α) is a major member of the interleukin-1 (IL-1) family, which has been well established as pro-inflammatory cytokine cluster involved in tumorigenesis. Several studies have showed that IL-1α contributes to tumour invasion, tumour proliferation, tumour metastases and interaction between the host immune system and malignant tumour cells [9, 19]. Kasza has reported that IL-1α serves as a pleiotropic cytokine in human cancer progression as well as various immune and inflammatory responses, and the secreted IL-1α is an active form which induces the tissue damage and tumour growth together with other regulatory factors [15]. Accordingly, it is biologically reasonable that functional *IL1A* polymorphisms may play causal roles in the development of cancer.

Increasing evidence has showed that *IL1A* polymorphisms are associated with several cancers [14]. Fei Wang et al. has found that four *IL1A* SNPs, rs3783550, rs3783546, rs1609682, and rs3783521, were associated with a significantly increased risk of renal cell carcinoma [26]. Xiaofeng Zeng et al. has demonstrated the relationship between the *IL1A* rs3783553 polymorphism and a significantly decreased risk of gastric cancer (OR = 0.48, 95% CI:0.26–0.90, *p* = 0.02) [31]. Abazis-Stamboulieh D et al. has proved that the *IL1A* rs1800587 polymorphism was related to a higher risk of multiple myeloma development [1]. Our data showed that rs2856838 was significantly associated with CRC susceptibility (*p* = 0.036). After stratification by gender, we found that the recessive model of rs3783550, rs2856838, rs1609682, and rs3783521 were associated with increased CRC risk in females. Moreover, it should be noted that the incidence rate of CRC differs significantly according to gender [17]. Considering the predicted functions of the selected SNPs in our study and their influences on gene expression provided by database, we speculated that SNPs might affect oncogenic processes by altering gene expression and this process could be influenced by individual gender background, thus leading to different outcomes on CRC risk between males and females. Significant *IL1A* variants rs3783550, rs2856838, rs1609682 were located in the intron, and rs3783521 were in promoter. As both the intron and promoter play important roles in gene expression, the alterations of the SNPs might also influence gene via modulating the functions of these regions. In addition, gender may reflect the different levels of exposure to risk factors related to diet, occupational exposure, or lifestyle. However, due to the limitations of the collected data, it was only possible to study the effect of gender rather than the complete impacts of other factors contributing to the gene environment. Therefore, it is necessary to carry out a complete analysis on the interaction of these factors with genetic polymorphisms in CRC susceptibility to verify the results of this study.

Several limitations should be considered in this research. Firstly, the influence of the selected SNPs on *IL1A* gene expression did not verify in our population and the underlying mechanism upon this regulatory process still need to be further elucidated. Further research will be design to investigate the influence of the promising SNPs on intron and promoter functions in gene regulation, and the gender will be considered as an important factor in future research. Secondly, although variations of age and gender were considered during our analysis, some living habits were not evaluated in this study, such as alcohol consumption and cigarette smoking, which might facilitate the progression of CRC.

## Conclusion

Our study first provided objective evidence that the *IL1A* genetic polymorphism rs2856838 might contribute to the risk of CRC in Chinese Han population. In addition, rs3783550, rs1609682, rs3783521, and rs2856838 were susceptible pathogenic variants for CRC among females. These results could suggest new targets for CRC risk assessment, prevention and prognosis.

## Additional file

Additional file 1: **Table S1.** Primers used for identification of the IL1A polymorphisms. **Table S2.** Distributions of age and gender in CRC patients and controls. **Table S3.** Functional annotation of the selected variants provided by HaploReg 4.1. **Table S4.** eQTL analysis for the five IL1A SNPs provided by GTEx database. (DOCX 24 kb)

## Abbreviations

CI: Confidence interval
CRC: Colorectal cancer
eQTL: Expression quantitative trait loci
GTEx: Genotype-Tissue expression; SD: Standard deviation
HWE: Hardy-Weinberg equilibrium
Ins/del: Insertion/deletion
LD: Linkage disequilibrium
MAF: Minor allele frequency
MALDI-TOF mass spectrometry: Matrix-assisted laser desorption/ionization—time of flight mass spectrometry
OR: Odds ratio
SNPs: Single nucleotide polymorphisms
