# Supplementary material for: IL1A polymorphisms is a risk factor for colorectal cancer in Chinese Han population: a case control study
Source: BMC Cancer. 2019 Feb 28;19:181. doi: 10.1186/s12885-019-5395-9 (PMC6394039; doi:10.1186/s12885-019-5395-9)
Supplement: Supplementary file 1 — Table S1. Primers used for identification of the IL1A polymorphisms. Table S2. Distributions of age and gender in CRC patients and controls. Table S3. Functional annotation of the selected variants provided by HaploReg 4.1. Table S4. eQTL analysis for the five IL1A SNPs provided by GTEx database. (DOCX 24 kb) [file 12885_2019_5395_MOESM1_ESM.docx]

Supplementary Table S1 Primers used for identification of the *IL1A* polymorphisms

| SNP_ID | First PCRP (5'-3') | Second PCRP (5'-3') | UEP (5'-3') |
| --- | --- | --- | --- |
| rs3783550 | ACGTTGGATGTGAAGGCCAAATGCTAAGGG | ACGTTGGATGCTCAGGCATCTCCTATGAAG | CGAATTCTGTTAGAGAACAAGATG |
| rs3783546 | ACGTTGGATGACACTGCTGTTGGCACTATG | ACGTTGGATGGTAGGGCAGTAGCTTCATTC | CCTTATTCACTGAGAGCCTT |
| rs2856838 | ACGTTGGATGCTGGTGTCAGAGAAGACAAC | ACGTTGGATGGTTAGTTATGCCATCCTGAG | CCAGGTGTCTGTCTCCTAA |
| rs1609682 | ACGTTGGATGCATGGGACTGCTATTCTTAC | ACGTTGGATGACCTCTAGTGAGGGTAAAAC | GAGTGAGGGTAAAACAAAAGTATT |
| rs3783521 | ACGTTGGATGCCAACTGGCTACATTTCTGC | ACGTTGGATGTCAGGAGGAGAGGGTTAATC | CCTGTTGCCTAAAGAGGAA |

**SNP: Single nucleotide polymorphism; PCRP: PCR primer; UEP: Unique base extension primer.**

Supplementary Table S2 Distributions of age and gender in CRC patients and controls

| Variable | Case | % | Control | % | | *p* value |  |
| --- | --- | --- | --- | --- | --- | --- | --- |
| Total | 248 |  | 463 |  | |  |  |
| Sex |  |  |  |  | | 0.913^a^ |  |
| Female | 105 | 42.3 | 198 | 42.8 | |  |  |
| Male | 143 | 57.7 | 265 | 57.2 | |  |  |
| Age |  |  |  |  | | < 0.001^b^ |  |
| Mean ± SD | 58.69 ± 12.77 | | 50.65 ± 11.79 | |  | | |

**SD:** **Standard deviation.**

**^a^*p* value was calculated with Pearson's χ^2^ test.**

**^b^*p* value was calculated with independent samples *t*-test.**

Supplementary Table S3 Functional annotation of the selected variants provided by HaploReg 4.1

| SNP_ID | Ref/Alt | AFR freq | AMR freq | ASN freq | EUR freq | DNAse | Proteins bound | Motifs changed | dbSNP func annot |
| --- | --- | --- | --- | --- | --- | --- | --- | --- | --- |
| rs3783550 | G/T | 0.77 | 0.55 | 0.30 | 0.68 | SKIN, HRT, SKIN | / | Rad21, YY1 | intronic |
| rs3783546 | G/C | 0.77 | 0.55 | 0.30 | 0.68 | 6 tissues | POL2B | SIX5 | intronic |
| rs2856838 | G/A | 0.36 | 0.29 | 0.22 | 0.38 | BLD, SKIN, BLD | PU1, TCF12 | RREB-1 | intronic |
| rs1609682 | G/T | 0.77 | 0.55 | 0.30 | 0.68 | 5 tissues | BATF, EBF1 | Barhl1, Cdx, Sox | intronic |
| rs3783521 | G/A | 0.04 | 0.44 | 0.70 | 0.32 | BLD, BLD | / | 4 altered motifs | Promoter |

**SNP: Single nucleotide polymorphism; Ref: Reference; Alt: Alternation; AFR: African; AMR: American; ASN: Asian; EUR: European.**

Supplementary Table S4 eQTL analysis for the five *IL1A* SNPs provided by GTEx database

| SNP_ID | *p*-value | Normalized Effect Size | Tissue |
| --- | --- | --- | --- |
| rs3783550 | 1.9e-7 | 0.30 | Skin-Sun Exposed (Lower leg) |
|  | 0.0000015 | 0.30 | Skin-Not Sun Exposed (Suprapubic) |
|  | 0.000017 | 0.22 | Testis |
|  |  |  |  |
| rs3783546 | 1.9e-7 | 0.30 | Skin-Sun Exposed (Lower leg) |
|  | 0.0000015 | 0.30 | Skin-Not Sun Exposed (Suprapubic) |
|  | 0.000017 | 0.22 | Testis |
|  |  |  |  |
| rs2856838 | 5.6e-11 | 0.36 | Skin-Sun Exposed (Lower leg) |
|  | 5.8e-11 | 0.40 | Skin-Not Sun Exposed (Suprapubic) |
|  | 0.000045 | 0.20 | Testis |
|  |  |  |  |
| rs1609682 | 1.9e-7 | 0.30 | Skin-Sun Exposed (Lower leg) |
|  | 0.0000015 | 0.30 | Skin-Not Sun Exposed (Suprapubic) |
|  | 0.000017 | 0.22 | Testis |
|  |  |  |  |
| rs3783521 | 1.5e-8 | -0.33 | Skin-Sun Exposed (Lower leg) |
|  | 0.0000071 | -0.30 | Skin-Not Sun Exposed (Suprapubic) |
|  | 0.000018 | -0.42 | Pituitary |

**SNP: Single nucleotide polymorphism; eQTL: Expression quantitative trait loci.**
